# Inefficacy of dexamethasone and pirfenidone as comparators in the bleomycin-induced pulmonary fibrosis model in SD rats

**DOI:** 10.3389/fmed.2026.1822289

**Published:** 2026-05-11

**Authors:** Elena A. Tukhovskaya, Yulia A. Palikova, Maria S. Severyukhina, Alina M. Ismailova, Victor A. Palikov, Gulsara A. Slashcheva, Natalya A. Borozdina, Evgeniy S. Mikhaylov, Irina N. Kravchenko, Vitaly A. Kazakov, Ekaterina N. Kazakova, Elena A. Kalabina, Ekaterina A. Rasskazova, Maksim V. Shinelev, Elena S. Sadovnikova, Natalia F. Perepechenova, Vladimir A. Rykov, Karina A. Ivanova, Anna A. Kudriaeva, Alexander A. Globenko, Andrey S. Kolosov, Ekaterina A. Jain, Olga I. Patsap, Michael A. Ignatyuk, Alexey A. Belogurov, Arkady N. Murashev

**Affiliations:** 1 Branch of Shemyakin and Ovchinnikov Institute of Bioorganic Chemistry, Russian Academy of Sciences, Pushchino, Russia; 2 Shemyakin and Ovchinnikov Institute of Bioorganic Chemistry, Russian Academy of Sciences, Moscow, Russia; 3 Moscow Center for Advanced Studies, Moscow, Russia; 4 Valenta Pharm JSC, Moscow, Russia; 5 Peoples' Friendship University of Russia named after Patrice Lumumba, Moscow, Russia; 6 Department of Biological Chemistry, Russian University of Medicine, Ministry of Health of Russian Federation, Moscow, Russia

**Keywords:** bleomycine, comparator drugs, dexamethasone, pirfenidone, pulmonary fibrosis model, rats

## Abstract

**Background:**

Pulmonary fibrosis (PF) is a severe disease leading to disability and death. It is important to search for novel therapeutic agents, for which a model of bleomycin-induced PF is used and pirfenidone and dexamethasone are used as comparators. The aim of the study was to establish the effect of comparators on model PF.

**Methods:**

We induced PF in male SD rats by intratracheal administration of 2.5 mg/kg bleomycin (BLM). Dexamethasone was administered intravenously (0.5 mg/kg, 7 days,), and pirfenidone was administered orally (50 mg/kg, 21 day). Clinical signs of health deviations, body weight, weight gain, food consumption and spirometry were monitored. The lung condition, cellular composition of the bronchoalveolar fluid (BALF), the lung concentration of hydroxyproline, blood biochemistry and the severity of fibrosis were analyzed 8 and 22 days post PF induction.

**Results:**

All animals administered BLM intratracheally developed pronounced signs of PF, including weight loss leading to pulmonary cachexia, deterioration in general condition, increased respiratory rate and decreased vital capacity, increased hydroxyproline levels in lung tissue, elevated level of neutrophils, lymphocytes, and pulmonary macrophages in the BALF. All of these features developed over time and were evident on day 8 and 22 of the study. Histological analysis revealed a pronounced inflammatory response on day 8 and a generalized fibrotic process on day 22. Dexamethasone and pirfenidone not only failed to improve clinical condition of animals, but even worsened it; one animal receiving pirfenidone died.

**Conclusion:**

Animals with PF treated with dexamethasone and pirfenidone showed no improvement compared to untreated animals. Although dexamethasone reduced hydroxyproline levels, this did not improve either the animals’ general condition or lung damage.

## 1 Introduction

Idiopathic pulmonary fibrosis (IPF) is a serious, progressive, and irreversible disease of the lungs that results in significant disability and mortality. In Europe, annual deaths from IPF are rising and exceed 17,000 (1), while globally, the incidence reaches up to 12 per 100,000 people since 2000 (2). The prognosis for individuals diagnosed with IPF remains poor, with an average five-year survival rate not exceeding 25% (3). The causes of IPF are diverse and multifaceted, and are often associated with exposure to external factors such as environmental pollutants, dust, gases, and toxic substances are recognized contributors (4–8), as well as certain medications such as antineoplastic drugs (e.g., rituximab) (9).

To facilitate the discovery of new treatments for IPF, researchers commonly utilize a bleomycin-induced PF model in laboratory rodents. In this model, bleomycin (BLM) is administered intratracheally as a solution, initiating acute lung inflammation. This inflammatory response triggers the release of cytokines and growth factors, which in turn increase fibroblast proliferation and differentiation into myofibroblasts. The resulting accumulation of collagen leads to the formation of fibrotic foci and a loss of lung function, similar to the pathogenesis of human fibrotizing lung disease (10–13). BLM is used in a range of doses for modeling PF, from 0.3 mg/kg (14) to 10 mg/kg (15). In the present study, a dose of 2.5 mg/kg was selected based on literature review and our previous experiments (16–19). This relatively low dose is sufficient to induce PF while allowing for potential correction by therapeutic agents at the stage of fibrosis development (Table 1a–d).

**Table 1:** Use of dexamethasone and pirfenidone in rodent studies with PF model.

Animal species, PF model, dose, route of administration of the modeling drug	Drug, dose, administration schedule	Role of the drug in the study	Effect	Reference
a.				
Mice C57Bl/6, BLM 5 mg/kg, single dose, intratracheally	Pirfenidone, 50 mg/kg, orally, 21 days	Comparator drug	Improved Ashcroft histology Decreased collagen content in the lungs Decreased ROS generation rate	(80)
Rats SD, Paraquat intraperitoneally/20 mg/kg single dose/	Pirfenidone, 200 mg/kg, orally, 14 days Pirfenidone, 20 mg/kg, inhalation, 14 days	Test drug	Improvement of histology qualitatively Increase in SOD and CAT activity Decrease in LPO Decrease in hydroxyproline content	(81)
Mice C57Bl/6, BLM, 5 mg/kg, intratracheally single dose	Pirfenidone 300 mg/kg, orally, 21 days	Test drug	No quantitative improvements in histology Reduction in IL6 and TNF-*α* in lung tissue	(34)
Mice C57Bl/6, BLM 3 mg/kg, single dose intratracheally	Pirfenidone, 30 mg/kg, orally, 21 days	Comparator drug	No improvement in weight loss Small improvement in forced vital capacity Improvement in quantitative histology Improvement in lung weight (decrease) Improvement in hydroxyproline content (decrease) Decrease in Myeloperoxidase activity Decrease in THF-α	(76)
Wistar rats, BLM, 5 mg/kg, intratracheally	Pirfenidone, 50 mg/kg, orally, 28 days	Comparator drug	Positive effects on body weight and lung weight Reduction in TNF-α and IL-6 in the lungs Reduction in collagen content in the lungs Reduction in damage in histological quantification according to the Ashcroft scale	(75)
Rats SD, BLM, 5 mg/kg, intratracheally	Pirfenidone, 100 mg/kg, orally, 21 days	Comparator drug	Only Masson-stained sections are shown, not semi-quantitatively	(83)
Rats SD, BLM, 10 mg/kg, intratracheally	Pirfenidone, 420 mg/kg, orally, 15 days	Comparator drug	Prevents lung weight gain Prevents weight loss Decreases TNF-α and IL-6 in the lungs Decreases hydroxyproline in the lungs Improves the PF pattern in the lungs—Ashcroft semi-quantitative assessment	(15)
Mice BALB/c, BLM, 4 mg/kg, intratracheally	Pirfenidone, 150 mg/kg, orally, 14 days	Comparator drug	Reduces lung weight relative to BLM Reduces hydroxyproline content in the lungs Reduces IL-6 and TGFβ content in the lungs	(77)
b.				
Wistar rats, BLM, 5 mg/kg, intratracheally	Pirfenidone, 100 mg/kg, PO, 15, 30, and 45 days	Test drug	Reduce pulmonary edema and improve the histological picture of PF	(82)
	Prednisone, 5 mg/kg, PO, 15, 30, and 45 days		Reduction in the content of PDFG, TNF-α and TGF-β1	
Wistar rats, BLM, 5 mg/kg, intratracheally	Priphenidone, 10, 30, 50, 100 mg/kg, orally, 7, 14 and 28 days	Test drug	The highest efficiency on histological preparations was demonstrated by a dose of 50 mg/kg Dose 50 mg/kg: reduces the severity of PF by histology, semi-quantitative assessment by Ashcroft Reduces the level of hydroxyproline in the lungs	(35)
Mice ICR, BLM, intravenously, 10 mg/kg, 5 days	Pirfenidone 10, 30, and 100 mg/kg, PO, 42 days	Test drugs	Pirfenidone at a dose of 100 mg/kg reduces histological expression semi-quantitatively according to Ashcroft Pirfenidone at doses of 30 and 100 mg/kg reduces the content of hydroxyproline in the lungs, the content of proinflammatory cytokines Reduces pulmonary edema	(33)
	Prednisolone 3 or 15 mg/kg, PO, 42 days		Prednisolone does not affect the content of hydroxyproline and the score according to Ashcroft Reduces pulmonary edema	
Wistar rats, BLM, 5 mg/kg, intratracheally	Pirfenidone 100 mg/kg orally, 28 days	Comparator drug	Reduces weight loss Reduces pulmonary edema Reduces Ashcroft fibrosis score semi-quantitatively Reduces hydroxyproline content Increases SOD activity	(84)
C57BL/6 mice, BLM, 5 mg/kg, intratracheally	Pirfenidone, 300 mg/kg, orally, 42 days	Comparator drug	Reduces Ashcroft fibrosis score semi-quantitatively Reduces inflammation score Reduces ROS generation in lung tissue Reduces MDA in blood and BALF	(85)
C57BL/6 J mice, BLM, 2 mg/kg, intratracheally	Pirfenidone, 200 mg/kg, orally, 7 and 14 days	Comparator drug	Reduces Ashcroft fibrosis score semi-quantitatively Reduces the number of lymphocytes, macrophages and neutrophils in the BALF Reduces the content of hydroxyproline	(86)
Rats, BLM, 5 mg/kg, intratracheally	Pirfenidone, 50 mg/kg, orally, 15 days	Comparator drug, test drug (in combination with the flavonoid fisetin)	In combination with fisetin: Reduces the cell content in the BALF Reduces the content of hydroxyproline	(36)
c.				
Rats, BLM, 5 mg/kg, intratracheally	Pirfenidone, 50 mg/kg, orally, 14 days	Comparator drug	Reduced lung weight Reduced hydroxyproline content Reduced neutrophil and leukocyte content in BALF	(37)
Wistar rats, BLM, 5 mg/kg, intratracheally	Dexamethasone, 0.5 mg/kg, orally, 28 days	Comparator drug	Semi-quantitative assessment of PF in histological preparations—no difference with the group without treatment Reduces the content of macrophages	(41)
Wistar rats, BLM, 5 mg/kg, intratracheally	Dexamethasone, 3 mg/kg, intraperitoneally, 14 days	Comparator drug	Reduces hydroxyproline levels in the lungs Reduces collagen I levels in the blood Reduces MMZ2 and MMP9 levels in the blood	(87)
Rats SD, BLM, 5 mg/kg, intratracheally	Dexamethasone, 5 mg/kg, intraperitoneally, 1, 3, 7, 14 and 28 days	Test drug	Reduces neutrophil levels in BAL Increases apoptotic index of inflammatory cells	(42)
Rats SD, BLM, 5 mg/kg, intratracheally	Dexamethasone, 5 mg/kg, intraperitoneally, 1, 3, 7, 14 and 28 days	Test drug	Causes death of animals up to 60% with prolonged use for 28 days Deterioration of condition with administration for 28 days Reduces the content of hydroxyproline in the lungs Reduces the content of neutrophils, lymphocytes and macrophages in the BALF Reduces the expression of mRNA of inflammatory and mitogenic mediators (TNF-α, TGF-β1, MCP-1, PDGF-β, endothelin-1 and GAPDH) Reduces the severity of PF in histological preparations—semi-quantitatively Increased the apoptotic index in the lungs, higher than BLM A conclusion is made about the harmfulness of long-term use	(88)
Wistar rats, BLM, 5 mg/kg, intratracheally	Dexamethasone, 4 mg/kg, intraperitoneally, 1, 14 and 28 days	Test drug	Didn’t improve any indicators	(79)
Male SD rats, BLM, 5 mg/kg, intratracheally	Dexamethasone, 3 mg/kg, 14 days	Comparator drug	Reduction of PF expression in histological preparations evaluated by Szapiel scoring method Reduction of hydroxyproline content	(89)
Female Wistar rats	Dexamethasone	Comparator drug	Reduced injury and fibrosis in lung tissue, of TNF-α and IL-6 Increases neutrophil, lymphocyte, and macrophage counts in BAL fluid	(90)
d.				
Male Lewis rats, BLM, 1.5 mg/kg, intratracheally	Dexamethasone, 0.5 mg/kg, intraperitoneally, 3 days (starting from the 3rd day after PF modeling)	Test drug	On the 14th day after PF modeling: Decreased % of macrophages in BALF Decreased collagen content in the lungs Decreased apoptosis of endothelial cells in the lungs 5 days after PF induction	(91)
Male Wistar rats, BLM, 5 mg/kg, intratracheally	Dexamethasone, 1 mg/kg, intraperitoneally, 14 days	Comparator drug	28 days after PF induction: Decreases hydroxyproline content Increases SOD, MDA Does not prevent weight loss Does not improve oxygen saturation	(92)
Male Wistar rats, methotrexate, 14 mg/kg, once weekly for 2 weeks, orally	Dexamethasone 0.5 mg/kg, orally for 7 days starting from the day of the last methotrexate administration	Comparator drug	At euthanasia 6 weeks after PF modeling: Increases SOD activity Decreases the percentage of fibrotic tissue Does not affect the content of collagen (hydroxyproline) in the lungs Does not affect the increase in IL-4 in the lungs Decreases the content of TGF-β1 in the lungs	(93)
C57BL/6 mice males, BLM, intratracheally, 2.5 mg/kg, 7 days	Desamethasone after 7-day of BLM administration: days 1–11—1 mg/kg, days 12–25—1.5 mg/kg	Comparator drugs	Reduces hydroxyproline in the lungs Reduces the severity of fibrosis in the lungs	(17)
	Pirfenidone, 200 mg/kg, PO 18 days after 7-day BLM administration		Does not improve weight loss Reduces the severity of PF (Ashcroft score) Reduces the content of leukocytes and lymphocytes in BAL	
Mice, BLM 1.5 mg/kg, intratracheally, 5 days	Dexamethasone, 3 mg/kg, intravenously, 15 days	Comparator drug	Does not improve weight loss Does not improve fibrosis (semi-quantitative assessment) Decreases pulmonary hydroxyproline concentrations	(40)
C57BL/6 mice, BLM, intratracheal	Dexamethasone, 0.5 mg/kg orally, 21 days	Combination with the drug under test—alfacalcidol	The combination, but not dexamethasone itself, reduces proinflammatory cytokines IL-1β, IL-6, TNF-α and TGF-β, as well as lung hydroxyproline levels	(38)
Female NMRI mice and Sprague–Dawley rats Silica, 0.5–5 mg in 60 μL into lungs of mice, 3 or 30 mg in 360 μL into lungs of rats	Dexamethasone, 2.5 μg/mL in drinking water starting 3 days before silicon instillation, 60–120 days	Test drug	Reduces lung collagen levels in SD rats but not NMRI mice	(78)
Male mice C57Bl/6 J, BLM, 3.75 mg/kg, intratracheally,	Dexamethasone, 2.5 mg/kg orally on days 1–14 (preventive protocol) or days 7–21 (therapeutic protocol)	Comparator drug	Preventive but not therapeutic administration results in decreased collagen mRNA expression	(39)

To validate the PF model and facilitate comparative efficacy studies, it is standard practice to use comparator drugs with known antifibrotic effects. Such comparators enable assessment of the model’s suitability and provide benchmarks for evaluating new candidate treatments. In many studies employing the BLM-induced PF model, pirfenidone and dexamethasone are commonly used as comparators (Table 1a–d). Pirfenidone is one of two drugs (alongside nintedanib) recommended by the FDA for PF treatment. In clinical settings, pirfenidone has been shown to improve lung function in humans (20–22), though it does not necessarily reverse fibrotic changes or enhance survival rates (23). The mechanism of action of pirfenidone is not fully understood, but it is associated with reduced production of certain proinflammatory cytokines, decreased transcription of profibrotic factors (such as TGF-β), diminished oxidative stress and less lipid peroxidation (24, 25).

Dexamethasone is a synthetic glucocorticosteroid widely used for treating acute inflammatory conditions, including multiple sclerosis exacerbations, allergies, cerebral edema, inflammation, and shock. Its mechanism involves suppression of neutrophil migration, reduction of lymphocyte proliferation, and inhibition of proinflammatory cytokines such as interleukin-1, -12, -18, tumor necrosis factor (TNF), interferon-gamma, and granulocyte-macrophage colony-stimulating factor (GM-CSF)^1^ (26). During the COVID-19 pandemic, dexamethasone was administered to patients requiring supplemental oxygen or ventilation, but not for those with mild forms of COVID 19 (27–29). Although corticosteroids are not standard therapy for PF (30), some pulmonologists employ short courses (3 day) of high-dose corticosteroids (500–1,000 mg), followed by a dose reduction, or pulse therapy for only 3 days (31, 32).

The primary aim of this study was to assess the efficacy of pirfenidone and dexamethasone as comparators in a BLM-induced PF model using male SD rats. The intention was to determine whether these drugs could serve as effective benchmarks for evaluating novel agents targeting PF. Dosage and administration regimens were selected based on those commonly used in previous rodent studies (Table 1). Thus, for mice, pirfenidone doses range from 10 mg/kg (33) to 300 mg/kg orally (34), while for rats, doses between 10 mg/kg (35) and 420 mg/kg (15) have been employed. In this study, rats received pirfenidone at 50 mg/kg orally, a dose previously shown to be more effective (35–37) than even higher doses (35). For dexamethasone, mouse studies have used oral doses between 0.5 mg/kg (38) and 2.5 mg/kg (39) and intravenous doses up to 3 mg/kg (40). In rats dexamethasone is used at doses of 0.5 mg/kg orally (41) to 5 mg/kg parenterally (42). Here, dexamethasone was administered parenterally at 0.5 mg/kg, with a short-term regimen reflecting clinical practice and minimizing the risk of immunosuppression associated with prolonged corticosteroid use (43, 44).

## 2 Materials and methods

### 2.1 Animals

The study used 48 young, sexually mature male SD rats that met the criteria for SPF (specific pathogen free) status, aged 9–10 weeks at the beginning of the study, with an average weight of 280 ± 15 g. Animals were obtained from the Unique Research Unit Bio-Model of the IBCh, RAS; the Bioresource Collection—Collection of SPF-Laboratory Rodents for Fundamental, Biomedical and Pharmacological Studies, no. 075-15-2025-486. All procedures and manipulations with animals were approved by Committee for Control over Care and Use of Laboratory Animals of BIBCh RAS (IACUC) (protocol number 946/24 from 15.02.2024) and were carried out in accordance with the EU Directive 2010/63/EU. After receiving from the nursery, the animals were adapted within 7 days. During the adaptation period, the health status of the animals was monitored through a clinical examination by assessing appearance and behavior of the animals. Animals with no signs of health problems were selected for the experiment. Animals were randomly divided into groups using body weight as a criterion so that the average weight of animals did not differ between groups. Each animal was assigned an individual number, according to which the animal was marked with a puncture of the auricle. During the study, the animals were kept under controlled environmental conditions in a barrier zone with a “clean” and “dirty” corridor system with controlled environmental conditions (temperature 20–24 °C, relative humidity 30–55%, 12 h light cycle; 08:00–20:00—“day,” 20:00–08:00—“night”; 10-fold change in air volume in the room per hour). The animals received ad libitum food for laboratory mice and rats Velaz FORTI 1324 Maintenance Diet (Altromin Spezialfutter GmbH & Co KG, Im Seelenkamp 20, D-32791 Lage, Germany).

### 2.2 Brief description of the design

The animals were divided into 4 groups of 12 animals (Table 2). PF modeling in animals was performed by intratracheal administration of BLM at a dose of 2.5 mg/kg. The control group was administered physiological saline intratracheally. Volume of intratracheal injection was 0.5 mL/kg. Animals of groups 1and 2 were administered vehicle—Tween 80 orally by gavage once a day for 21 days, animals of group 3 were administered dexamethasone at a dose of 5 mg/kg intravenously in the tail vein once a day for 7 days, and animals of group 4 were administered pirfenidone at a dose of 50 mg/kg once a day by gavage for 21 days. The first administration of all drugs was performed 1 h before PF modeling. Animals were recorded weekly for body weight and weight gain, food consumption, respiratory function (spirometry), and clinical signs of health abnormalities. Half of the animals were euthanized on day 8 after PF modeling to assess the state during the inflammatory phase of PF formation, and other half were euthanized on day 22 after intratracheal administration of BLM to assess the formed PF. During necropsy, lungs were removed, BAL was performed, and blood was collected to obtain serum. Lungs were weighed, taken for hydroxyproline ELISA, visually assessed, fixed, and semiquantitative histological assessment was performed in histological preparations of the lungs stained with Masson’s trichrome.

**Table 2:** Groups and doses.

Group number	Modeling	Drug, dose	Duration of administration	Route/volume of drug administration	Animal numbers	
					Euthanasia Day 8	Euthanasia Day 22
1	Saline solution, intratracheally in a volume of 0.5 mL/kg	Twin 80 0.25%	7 days	Orally by gavage, 5 mL/kg	1–6	–
			21 days		–	7–12
2	BLM, 2.5 mg/kg, intratracheally in a volume of 0.5 mL/kg	Twin 80 0.25%	7 days	Orally by gavage, 5 mL/kg	13–18	–
			21 days		–	19–24
3		Dexamethasone 0.5 mg/kg	7 days	Intravenously, 5 mL/kg	25–30	31–36
4		Pirfenidone 50 mg/kg	7 days	Orally by gavage as a suspension in Tween 80, 5 mL/kg	37–42	–
			21 days		–	43–48

### 2.3 PF modeling

The procedure for modeling PF was performed as described previously (45). Animals from group 1 were administered saline intratracheally, all other animals (groups 2–4) were administered BLM solution intratracheally at a dose of 2.5 mg/kg, in a volume of 0.5 mL/kg in an anesthetized state using a tube with a round tip, through which a thin catheter with a syringe with a BLM solution attached was threaded. The introduction was carried out by bolus, by injecting BLM solution into the trachea in a volume of 0.5 mL/kg. Immediately after this, the animals were connected to a ventilator (Ugo Basile) and subjected to hyperventilation of the lungs (parameters: tidal volume of the lungs—30 mL/kg, respiratory rate 60 times per minute, exposure time 10 min).

### 2.4 Drugs

To model PF, we used the pharmaceutical preparation bleomycin hydrochloride manufactured by JSC Omutninskaya Scientific Experimental Industrial Base, Russia. Dexamethasone in 1 mL ampoules of 4 mg/1 mL dexamethasone solution, Elfa Laboratories, India, and Pirfenidone in the form of Esbriet tablets, film-coated tablets (Pirfenidone 267 mg/tablet), Delpharm Milano S.r.l. were used as comparison drugs. For the administration of pirfenidone, Esbriet tablets were crushed in a mortar and mixed with the Tween 80 vehicle to form a suspension. Tween 80 (Tween 80 BioChemica), PanReac AppliChem ITW Reagenst, GmbH, Germany, was used as a carrier for preparing the pirfenidone suspension and as a control drug. Control animals from the first group were administered intratracheal saline solution produced by JSC Dalhimfarm, Russia. To anesthetize animals during the PF modeling and before necropsy, we used Telazol, Zoetis Manufacturing & Research Spain, S.L., Spain, mixed with Xyla, Interchemie werken “De Adelaar” BV, Estonia.

### 2.5 Lifetime observations

#### 2.5.1 Registration of mortality and signs of deviation in the health of animals

The health status of the animals was assessed before group formation and on days 3, 7, 14 and 21 after PF modeling. In the presence of significant deviations in health, the health status of the animals was recorded daily. All animals were observed in their cages twice daily (morning and afternoon) to detect severe conditions and mortality. Animals were also observed for approximately 1 h after dosing to detect administration-related abnormalities (toxicity, trauma, aspiration, etc.). When abnormalities were detected, they were recorded on a special sheet and, at the end of the study, were assessed according to the parameters listed in Table 3. Deviations in the health status of animals were also assessed according to the above-listed features, determining the number of animals with the presence of the features and the total number of animals in the group over a time period of 7 days, namely on days 1–7, 8–15 and 16–21.

**Table 3:** List of animal health abnormalities.

Description of the feature/presence or duration		Score
Wheezing	Absent	0
	Present	1
Weight loss	Absent	0
	Duration up to 5 days	1
	Duration from 5 to 8 days	2
	Duration up to 8–15 days (for animals undergoing necropsy on the 22nd day)	3
	Duration over 15 days (for animals necropsied on day 22)	4
Tachypnoe	Absent	0
	Duration up to 5 days	1
	Duration from 5 to 8 days	2
	Duration up to 8–15 days (for animals undergoing necropsy on the 22nd day)	3
	Duration over 15 days (for animals necropsied on day 22)	4
Eye/nasal discharge	Absent	0
	Present	1
Retracted abdomen	Absent	0
	Present	1

#### 2.5.2 Body weight and weight gain

Body weight was recorded daily. Weight gain was calculated as the increase in body weight relative to body weight on the first day of the study (PF modeling day).

#### 2.5.3 Food consumption

Food consumption was recorded before PF modeling, then on the 2nd–3rd day after modeling, and then weekly, namely on the 6th–7th, 13th–14th, 20th–21st days after PF modeling.

#### 2.5.4 Assessment of external respiratory function (spirometry)

Respiratory parameters (respiratory rate, tidal volume, and maximum expiratory volume) were recorded before administration on day 0, then on days 2, 7, 14, and 21 of the study in animals using an FE141 Spirometer on a PowerLab 8/35 computer system (ADInstruments Pty Ltd., Australia).

### 2.6 Euthanasia and necropsy

Euthanasia was performed by anesthesia with a mixture of Telazol® (tiletamine + zolazepam) + Xyla in doses of 40 mg/kg + 10 mg/kg, followed by total blood sampling from the inferior vena cava. During necropsy, blood samples was taken for biochemical analysis and the animal lungs and trachea were removed, clearing them of connective tissue. Half of the animals were euthanized on day 8 and the other half on day 22 after PF modeling.

### 2.7 Lung weight

During necropsy, the lungs with trachea were weighed and the relative weight of the lungs relative to body weight was calculated.

### 2.8 Visual semiquantitative assessment of the lungs at necropsy

At necropsy, after extraction, cleaning and washing of blood, the lungs were photographed from both sides—ventral and dorsal, placing them on a white background. The visual assessment criteria given below were developed, using which the appropriate score was assigned to the lungs (Table 4). Visual scoring of the lungs was performed by two independent researchers, after which the scores assigned by them were averaged.

**Table 4:** Criteria for visual semiquantitative assessment of lung damage.

Description of the criterion	Presence/expression	Score	Photo with example	Description of the criterion	Presence/expression	Score	Photo with example	Description of the criterion	Presence/expression	Score	Photo with example
Swelling of the left or right lung	Absent	0	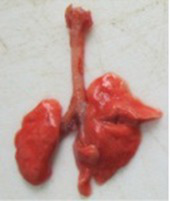 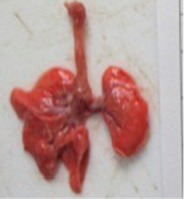	Presence of hemorrhages	Absent	0	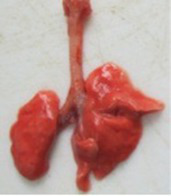 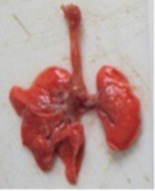	Dark discoloration in the left or right lung	Absent	0	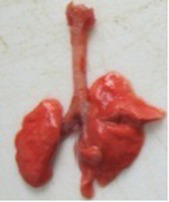 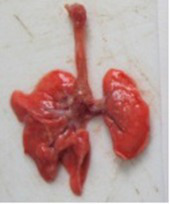
	Present	1	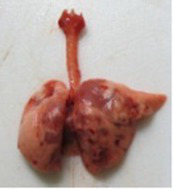 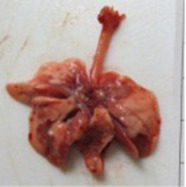 Left lung swelled		From 1 to 3	1	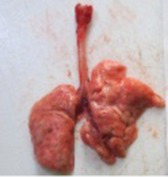 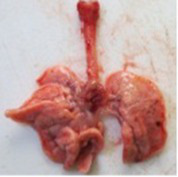		Area within 10%	1	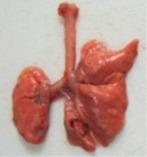 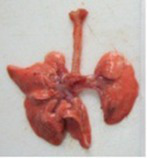
	Present	1	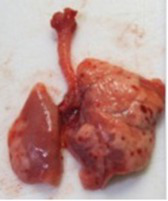 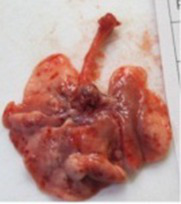 Right lung swelled		From 3 to 10	2	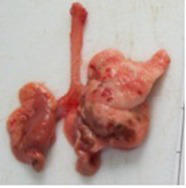 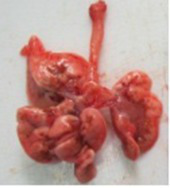		Area from 10 to 25%	2	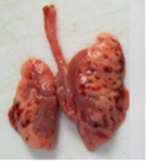 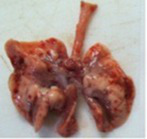
Decrease or enlargement of one or both lungs	Absent	0	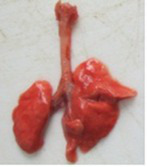 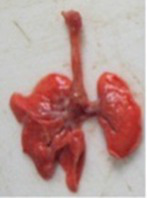		Over 10	3	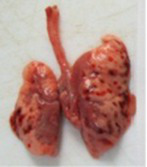 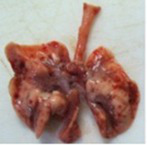		Area over 25%	3	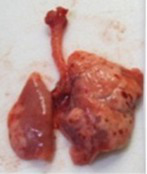 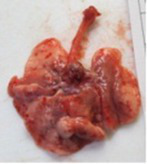
	Present	1	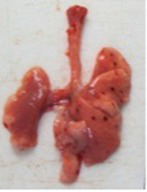 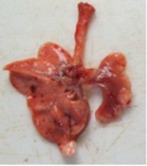 The left lung is reduced	General change in lung color to light (focal or diffuse) compared to control	Absent	0	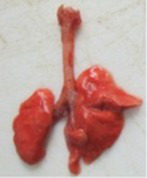 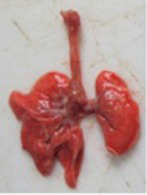	Lung deformation	Absent	0	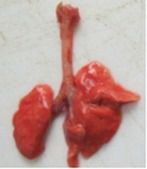 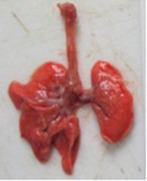
	Present	1	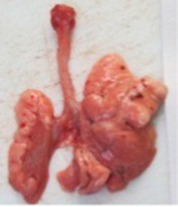 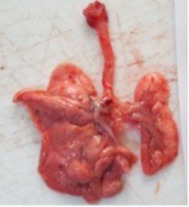 The right lung is enlarged		Up to 25%	1	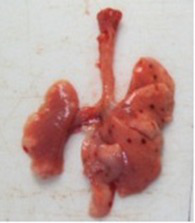 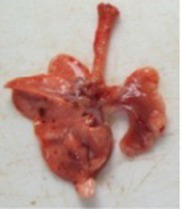		Present	1	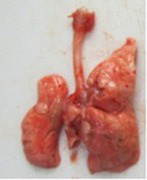 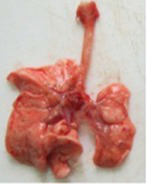
					Over 25%	2	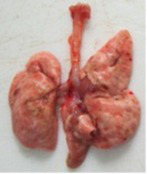 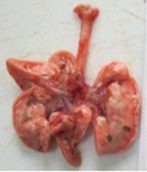				

### 2.9 BAL

The right bronchus was ligated and BAL was performed by introducing 2 mL of PBS solution into the lung through the trachea and then draining the resulting wash by gravity into a 15 mL test tube. The procedure was performed three times, with a total volume of PBS for BAL of 6 mL. The resulting BALF was divided into two parts, one of which was transferred for counting the total number of cells in the wash in a Goryaev chamber, and the second for centrifugation and subsequent preparation of a smear to determine the cellular composition per 100 cells.

### 2.10 BALF analysis

Half of the BALF was taken to calculate the concentration of nucleated cells. For this purpose, a tenfold dilution of the obtained wash was performed in 4% acetic acid to lyse the erythrocytes, the resulting sample was taken, the Goryaev chamber was filled and the nucleated cells were counted. The second half of the BALF was taken to prepare a smear with subsequent cell counting. The BALF was centrifuged in an Eppendorf 5804 R centrifuge at 3,000 rpm, 4 °C for 15 min. The supernatant was collected and a smear was prepared from the sediment after pipetting with 3 μL of fetal calf serum. The smear was stained according to Pappenheim. The cellular composition of stained smears was analyzed: the percentage of alveolar macrophages, segmented neutrophils, band neutrophils, eosinophils, lymphocytes, and mast cells was calculated per 100 cells.

### 2.11 Lung sampling and sample preparation for hydroxyproline ELISA and analysis

After performing BAL for the left lung, the left bronchus was ligated and the left lung was excised. The left lung was divided into two parts, which were placed in weighed 2 mL Eppendorf tubes. The lung samples were frozen at −70 °C until homogenate preparation. After defrosting, the lung samples were homogenized with a manual homogenizer MT-30 K, the homogenate was weighed, frozen at −70 °C, defrosted, PBS solution was added to the homogenate in a ratio of 1:12 (weight:volume), mixed. The homogenate + PBS was frozen at −70 °C, defrosted, frozen again, then defrosted and centrifuged at 3,000 rpm, 4 °C, 15 min, and then immediately centrifuged again at 5,000 rpm, 15 °C, 15 min. The tubes for concentration were pre-washed with PBS solution according to the instructions. The supernatant from the homogenate + PBS mixture was poured into the washed tube and centrifuged at 3,500 rpm, 21 °C. The centrifugation time for all samples was different (from 30 to 140 min). The samples were concentrated to a two-fold volume relative to homogenate weight (e.g., homogenate weight 0.3 g—centrifugation to a volume of 0.6 mL).

### 2.12 Procedure for analysis of left lung homogenate by ELISA

Hydroxyproline concentration in samples obtained from left lung homogenate was determined using the Rat Hyp (Hydroxyproline) ELISA Kit, ELK Biotechnology, China.

### 2.13 Analysis of biochemical parameters

Serum was obtained from blood collected from the inferior vena cava during necropsy by centrifugation at 1,600 g, 4 °C, 15 min. The obtained serum was frozen at −20 °C until analysis. Serum samples were analyzed on a SAPPHIRE 400 automated biochemical analyzer (Prestige 24i, Tokyo Boeki, Japan) using reagents from Randox Laboratories Ltd. according to the parameters listed in Table 5.

**Table 5:** List of biochemical parameters measured in animal serum.

Total protein	Creatinine
Albumin (A)	Urea
Globulin (G)—calculated	Total bilirubin
A/G ratio—calculated	Total cholesterol
Alkaline phosphatase (ALP)	Triglycerides
Aspartate aminotransferase (AST)	Calcium
Alanine aminotransferase (ALT)	Inorganic phosphates

### 2.14 Histology of the right lung

The right lung, after cutting off the left lung and ligating the left bronchus through the trachea, was filled with 10% neutral formalin and placed in this fixative for at least 48 h, after which it was washed in running tap water, dehydrated in alcohols of increasing concentration and embedded in paraffin. Paraffin sections 4–5 μm thick were stained with hematoxylin and eosin and according to the Masson method (Masson Trichrome dye, Bio-Optica, Italy). Histological preparations were studied using conventional light microscopy on an AxioScope. A1 microscope (Carl Zeiss, Germany). Micrographs of histological preparations were obtained using a high-resolution Axiocam 305 color camera (Carl Zeiss, Germany) using ZEN 2.6 lite software (Carl Zeiss, Germany). The severity of pathohistological changes in the lungs was assessed (1) using the generally accepted 5-point semi-quantitative scale in toxicological studies on histological preparations stained with hematoxylin and eosin (46), and (2) using the modified Ashcroft semi-quantitative 8-point scale (47) on paraffin sections stained using the Masson method. The histologist-pathologist who performed semi-quantitative assessment of lung preparations was “blinded” and unaware of group assignment of animals.

### 2.15 Statistics

Microsoft Excel and Statistica for Windows, version 7, were used for statistical processing of the experimental data. Between-group differences in repeated daily and weekly measures (body weight, weight gain, food consumption, spirometry) were assessed using repeated-measures analysis of variance ANOVA with power tested by Fisher’s LSD test. Between-group differences in single-item data (BALF cytology, lung tissue hydroxyproline concentration, lung weight, blood biochemistry results, and semiquantitative histological assessment data) were assessed using the Mann–Whitney U-test. The Chi-square test was used to analyze between-group differences in the frequency of trait manifestation (assessment of deviations in health status).

## 3 Results

### 3.1 Mortality and clinical signs of abnormalities in animal health

One animal receiving the comparator drug pirfenidone died on the 9th day after the PF modeling on the background of a rapid decrease in body weight (38%). At autopsy, massive hemorrhage into the lungs was observed. All animals with BLM-induced PF developed deviations in health associated with lung damage. During daily examination, starting from the 2nd day after PF modeling, the animals showed wheezing, increased respiratory rate, sunken sides, tremors, and weight loss with signs of dehydration. Table 6 presents the results of daily clinical observations, which show that in the first and second weeks after PF modeling, clinical signs indicating impaired pulmonary function develop equally in all animals that were intratracheally administered BLM. No positive effect of the comparison drugs—pirfenidone and dexamethasone—on the severity of deviations was observed. In the third week, no positive dynamics in the clinical manifestations of deviations is observed either. Moreover, the highest total number of animals with deviations is observed in the group that received the comparison drug dexamethasone. Table 7 presents the summary data of the semiquantitative scoring of the animals’ condition during daily observations. The highest score, indicating the greatest health abnormalities, was observed in animals treated with dexamethasone. Pirfenidone also demonstrated a high score of clinical signs by the end of observations. Therefore, both comparison drugs, pirfenidone and dexamethasone, do not improve the general condition of animals with the development of signs of lung damage, and even worsen it.

**Table 6:** Summary results of daily clinical observations.

	Number of animals with a trait from the total number of animals in the group			
	Group 1	Group 2	Group 3	Group 4
	Saline + Tween 80	BLM + Tween 80	BLM + dexamethasone	BLM + pirfenidone
Days 1–7				
	*N* = 12	*N* = 12	*N* = 12	*N* = 12
Wheezing	0/12	7/12*	2/12 ^@^	1/12 ^@^
Tachypnoe	0/12	9/12*	12/12*	11/12*
Retracted abdomen	0/12	8/12*	12/12*^@^	12/12*^@^
Weight loss	0/12	9/12*	12/12*	12/12*
Eye/nasal discharge	0/12	2/12	0/12	5/12*^&^
Total number of animals with deviations in the group	0/12	11/12*	12/12*	12/12*
Days 8–15				
	*N* = 6	*N* = 6	*N* = 6	*N* = 6
Death	0/6	0/6	0/6	1/6
Wheezing	0/6	0/6	0/6	0/6
Tachypnoe	0/6	6/6*	6/6*	6/6*
Retracted abdomen	0/6	5/6*	6/6*	6/6*
Weight loss	0/6	5/6*	6/6*	6/6*
Eye/nasal discharge	0/6	2/6	0/6	1/6
Unsteady gait	0/6	0/6	1/6	0/6
Total number of animals with deviations in the group	0/6	6/6*	6/6*	6/6*
Days 16–21				
	*N* = 6	*N* = 6	*N* = 6	*N* = 5^&^
Wheezing	0/6	0/6	0/6	0/5
Tachypnoe	0/6	2/6	5/6*	1/5
Retracted abdomen	0/6	2/6	4/6*	2/5
Weight loss	0/6	1/6	3/6	1/5
Eye/nasal discharge	0/6	1/6	0/6	0/5
Unsteady gait	0/6	0/6	1/6	0/5
Total number of animals with deviations in the group	0/6	2/6	5/6*	2/5

*p ≤ 0.05 relative to group 1, ^@^*p* ≤ 0.05 relative to group 2 according to Chi-square test; ^&^—in group 4 (BLM + pirfenidone) on the 9th day of the study, one animal died.

**Table 7:** Summary results of the scoring of the animals’ condition during daily clinical observations (assessment according to the parameters given in Table 1).

	Group 1		Group 2		Group 3		Group 4	
	Saline + Tween 80		BLM + Tween 80		BLM + dexamethasone		BLM + pirfenidone	
	Mean ± SD	*N*	Mean ± SD	*N*	Mean ± SD	*N*	Mean ± SD	*N*
Euthanasia on the 8th day								
Average score	0.0 ± 0.0	6	0.8 ± 0.8**	6	0.9 ± 0.8**	6	0.7 ± 0.6**	6
Euthanasia on the 22nd day								
Average score	0.0 ± 0.0	6	1.1 ± 0.9**	6	1.6 ± 1.6**	6	1.4 ± 1.4**	5^&^

** *p* ≤ 0.01 relative to group 1, which received Saline+Tween 80, in pairwise comparison using the Mann–Whitney U test; ^&^—in group 4 (BLM + pirfenidone), one animal died on the 9th day of the study.

### 3.2 Body weight and weight gain

**Figure 1:**
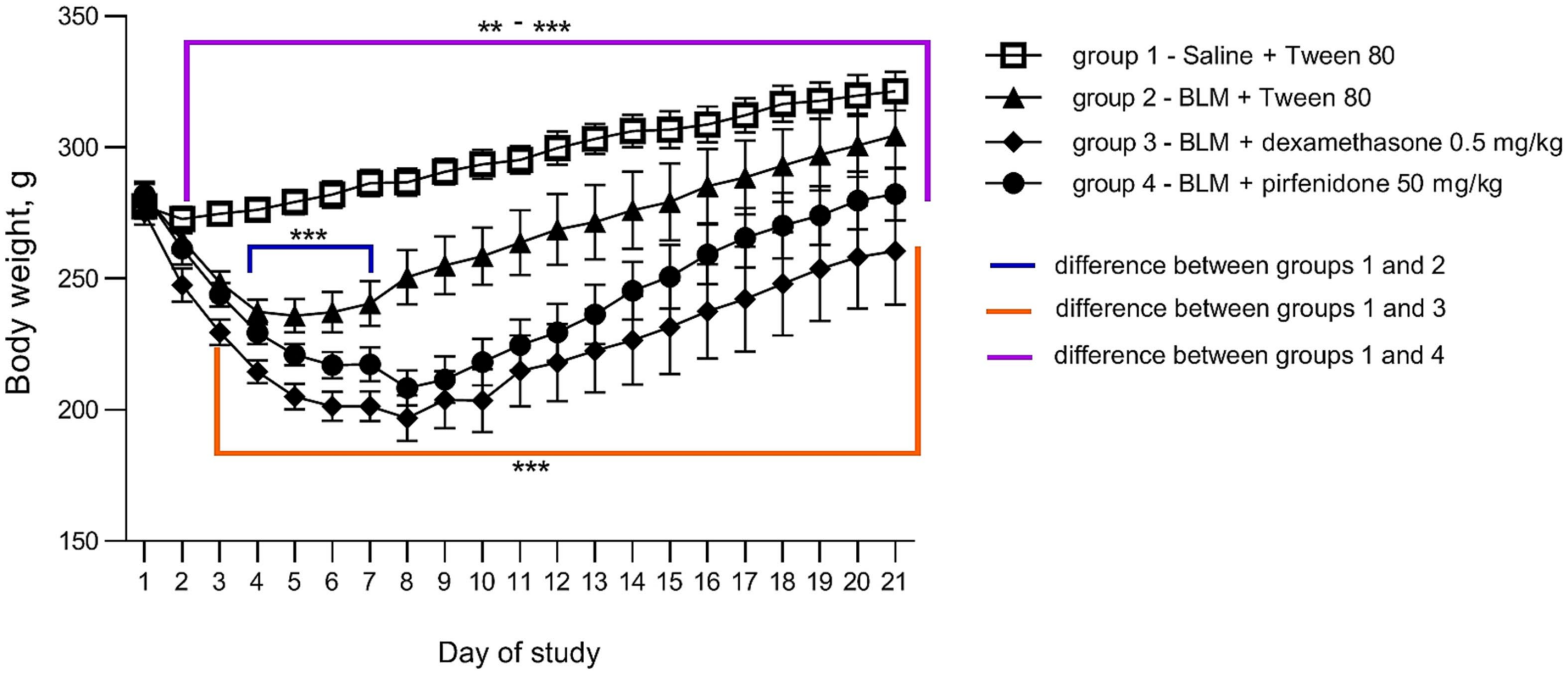
Dynamics of body weight changes post PF induction. **p ≤ 0.01, ***p ≤ 0.001 relative to group 1 saline + Tween 80 according to repeated measures ANOVA, post-hoc Fisher LSD test. In all groups up to the 7th day *n* = 12, on the 14th and 21st days *n* = 6, except for group 4 (BLM + pirfenidone), where one animal died on the 9th day of the study.

**Figure 2:**
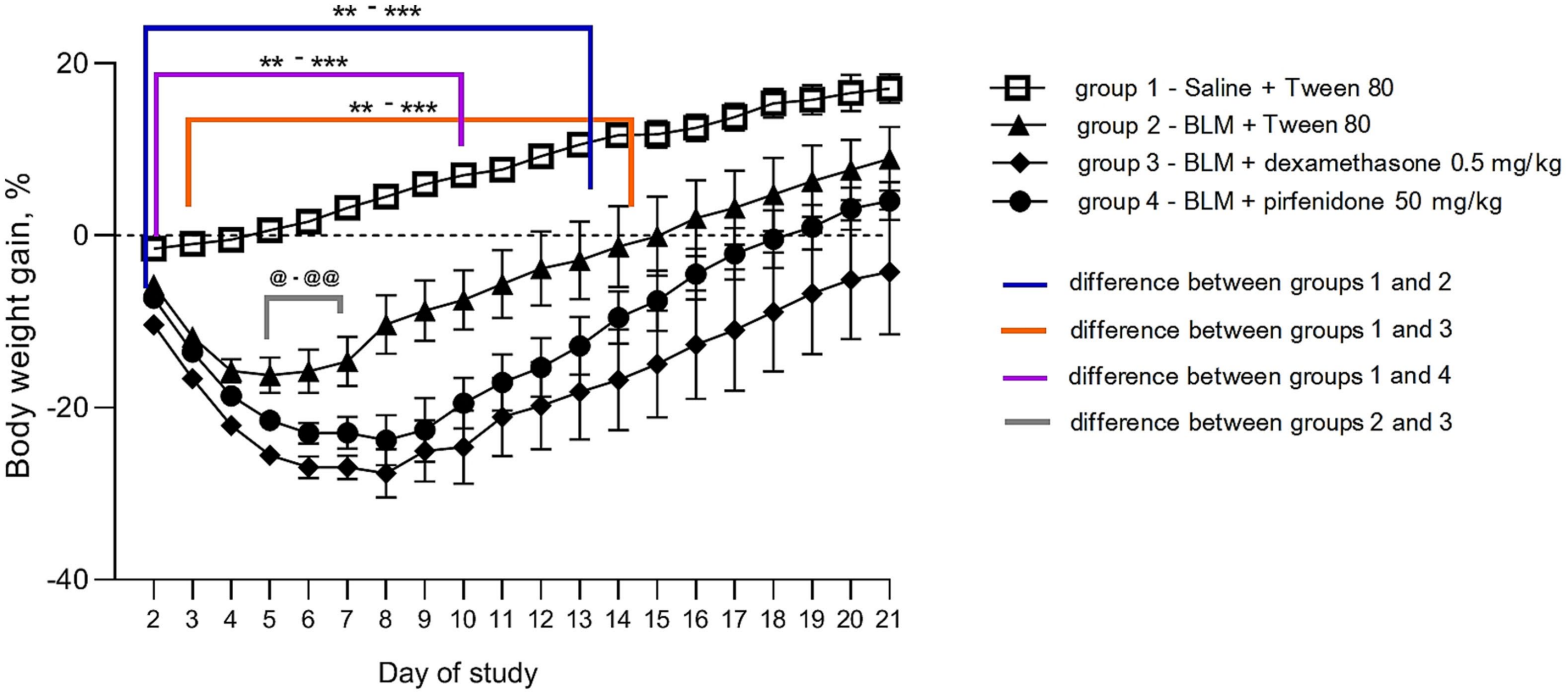
Dynamics of changes in body weight gain post PF induction. ^**^p ≤ 0.01, ^***^p ≤ 0.001 relative to group 1 saline + Tween 80, @ *p* ≤ 0.05, @@ *p* ≤ 0.01 relative to group 2 BLM + pirfenidone according to repeated measures ANOVA, post-hoc Fisher LSD test. In all groups up to the 7th day *n* = 12, on the 14th and 21st days *n* = 6, except for group 4 (BLM + pirfenidone), where one animal died on the 9th day of the study.

All animals with BLM-induced PF showed a decrease in body weight starting from the second day. However, in animals that received the comparison drugs dexamethasone and pirfenidone, this decrease persisted until the 21st day after induction, unlike non-treated animals, in which this indicator did not differ from that of the control animals after a week. The most pronounced decrease in body weight was observed in animals that received dexamethasone (Figure 1). In young healthy animals, there is a steady increase in body weight with age, as in the control group receiving saline + Tween 80. In non-treated animals decrease in the body weight was the most pronounced a week after modeling PF, after which a recovery started. In the groups that received the comparison drugs pirfenidone and dexamethasone, the body weight gain did not return to the baseline by the 21st day of observation, indicating, the comparison drugs worsened the general condition of the animals with PF (Figure 2).

### 3.3 Food consumption

**Figure 3:**
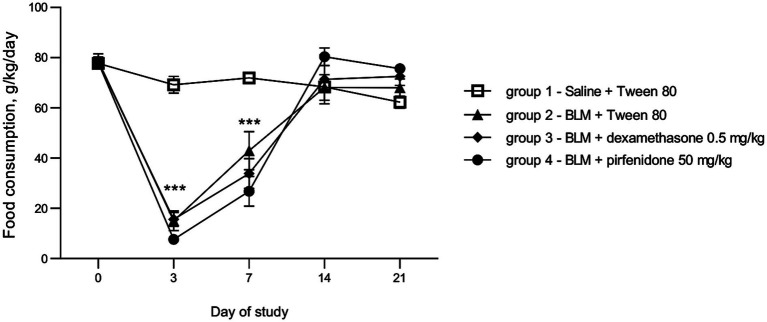
Dynamics of change in animal food consumption post PF induction. ***p ≤ 0.001 relative to group 1 saline + Tween 80 according to repeated measures ANOVA, post-hoc Fisher LSD test. In all groups up to day 7 *n* = 12, on days 14 and 21 *n* = 6, except for group 4 (BLM + pirfenidone), where one animal died on day 9 of the study.

Food consumption intake is one of the indicators of animal welfare. After PF induction, all animals showed a drop in feed intake on days 3 and 7 after modeling, followed by recovery by day 14. The administration of comparators dexamethasone and pirfenidone did not prevent the drop in food consumption (Figure 3).

### 3.4 Assessment of external respiratory function (spirometry)

**Figure 4:**
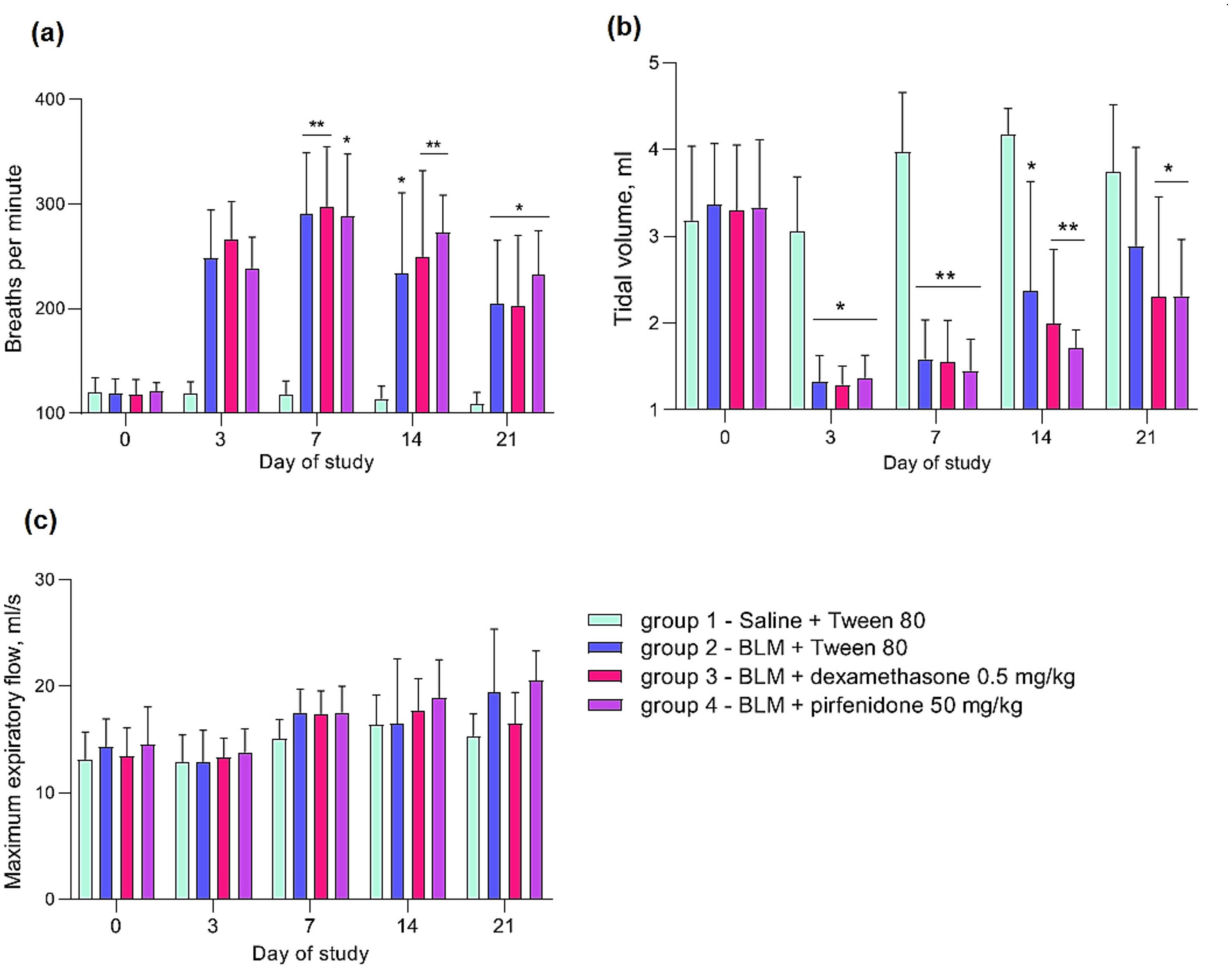
Dynamics of changes in external respiration parameters in rats post PF induction. **(a)** Respiratory rate; **(b)** tidal volume; **(c)** maximum expiratory flow. *p ≤ 0.05, **p ≤ 0.01, ***p ≤ 0.001 relative to group 1 saline + Tween 80 according to repeated measures ANOVA, post-hoc Fisher LSD test. In all groups up to the 7th day *n* = 12, on the 14th and 21st days *n* = 6, except for group 4 (BLM + pirfenidone), where one animal died on the 9th day of the study.

After PF induction in animals, there is a disruption of the external respiratory function, which is expressed in an increase in the respiratory rate and a decrease in the respiratory volume. The comparison drugs dexamethasone and pirfenidone did not improve, and even worsened the disruption of the respiratory function of animals with PF (Figure 4).

### 3.5 Macroscopic visual assessment of the lungs

**Figure 5:**
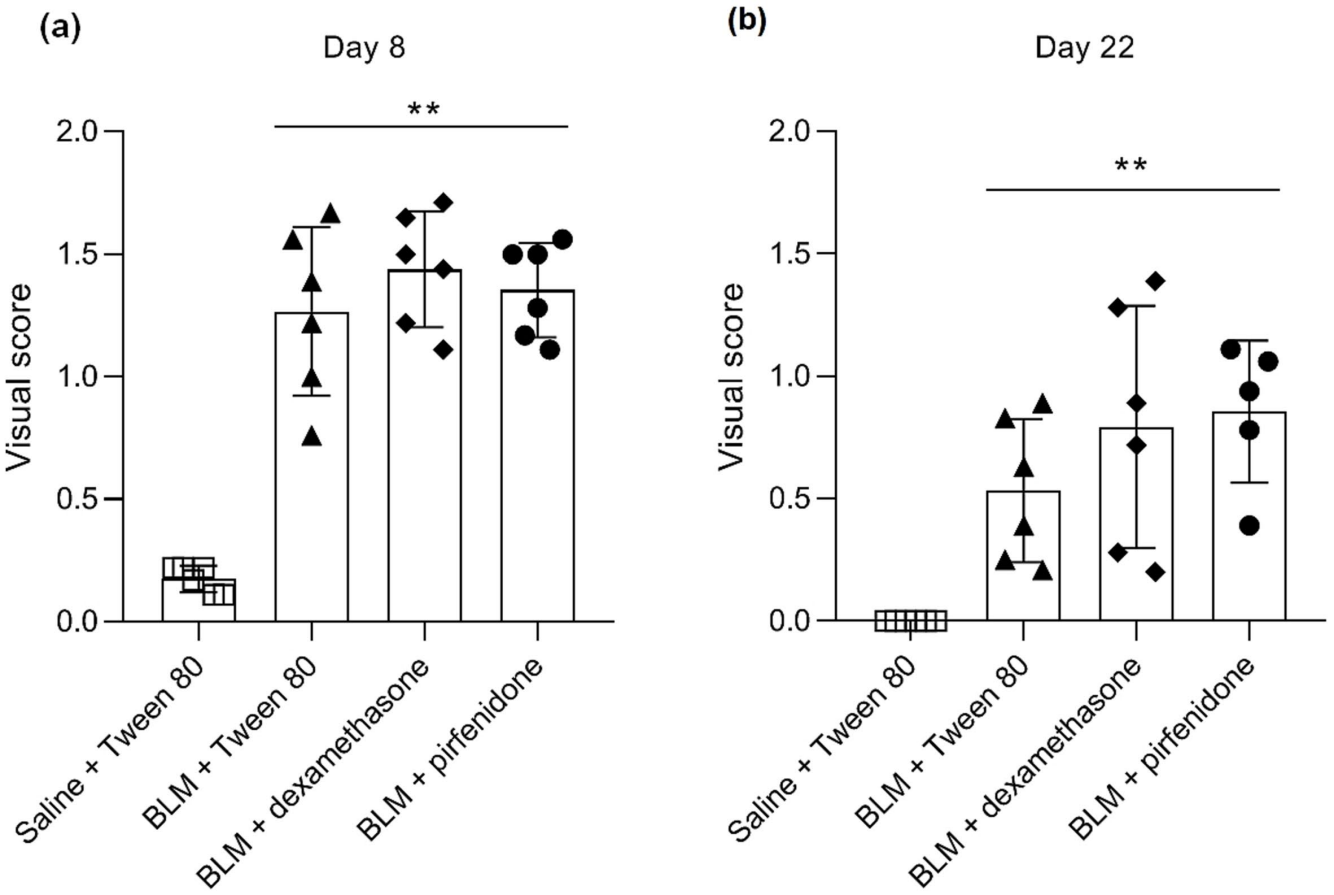
Semiquantitative visual assessment of the lungs at necropsy on day 8 **(a)** and day 22 **(b)**. ^**^ ≤ 0.01 relative to group 1 receiving saline + Tween 80, pairwise comparison using the Mann–Whitney *U* test. In all groups up to day 7 *n* = 12, on days 14 and 21 *n* = 6, except for group 4 (BLM + pirfenidone), where one animal died on day 9 of the study.

At necropsy on the 8th day of the study (7 days after the PF induction), the lungs of animals that received BLM intratracheally had an edematous appearance with large areas of tissue darkening and a change in the texture of these areas to a smoother one, with small multifocal hemorrhages. It should be noted that the lung damage of animals that received the comparison drugs dexamethasone and pirfenidone was similar in terms of severity compared to animals without treatment (BLM + Tween 80). Semiquantitative visual scoring of the lungs on the 8th day of the study revealed that the average score of lung damage was higher compared to that on the 22nd day due to the pronounced acute inflammatory process and associated edema. In the first group, which received intratracheal saline, inflammation was lower (0.18 ± 0.05 points), and in the groups that received intratracheal BLM to simulate PF, it ranged from 1.3 ± 0.3 in group 2 (BLM + Tween 80) to 1.4 ± 0.2 in group 3 (BLM + dexamethasone). At the same time, statistically significant difference between the groups receiving BLM were not observed. On the 22nd day of the study, the inflammatory process entered the chronic phase, its manifestations decreased, as a result of which the average lung damage score in the groups receiving BLM to model PF was lower compared to that on the 8th day and ranged from 0.5 ± 0.3 in group 2 (BLM + Tween 80) to 0.9 ± 0.3 in group 4 (BLM + pirfenidone). In the control group receiving intratracheal saline, the damage score was 0. Visually, lung damage was most pronounced in the all groups with PF including those received comparison drugs dexamethasone and pirfenidone (Figure 5).

### 3.6 Lung weight

In all animals given intratracheal BLM, lung weight, both absolute and relative (relative to body weight at necropsy), was increased relative to the control. Administration of the comparator drugs dexamethasone and pirfenidone did not affect this increase in lung weight (Table 8).

**Table 8:** Absolute and relative lung weight.

	Group 1	Group 2	Group 3	Group 4
	Saline + Tween 80	BLM + Tween 80	BLM + dexamethasone	BLM + pirfenidone
	Mean ± SD (*N* = 6)	Mean ± SD (*N* = 6)	Mean ± SD (*N* = 6)	Mean ± SD (*N* = 6)
Day 8				
Lung weight, g	1.6 ± 0.1	2.7 ± 0.5**	2.5 ± 0.4**	3.0 ± 4**
Relative lung weight, g/100 g body weight	0.6 ± 0.0	1.2 ± 0.4**	1.2 ± 0.2**	1.4 ± 0.4**
Day 22				
Lung weight, g	1.6 ± 0.1	2.4 ± 0.5**	2.4 ± 0.4**	2.8 ± 0.1**
Relative lung weight, g/100 g body weight	0.5 ± 0.0	0.8 ± 0.3**	1.0 ± 0.4**	1.0 ± 0.1**

** ≤ 0.01 relative to group 1, which received Saline + Tween 80, in paired comparison using the Mann–Whitney U test; &—in group 4 (BLM + pirfenidone), one animal died on the 9th day of the study.

### 3.7 BALF cytology

During necropsy on the 8th day, all animals with PF showed an increase in the concentration of cells in the BALF due to the elevated count of neutrophils, eosinophils, lymphocytes and monocytes, which is typical for the acute phase of lung damage and the inflammatory process. Changes were observed for all animals with PF, regardless of the administered drugs, in comparison with the control group 1, which received Saline + Tween 80. At necropsy on day 22, the concentration of all cells in the BALF remained equally increased in all groups due to the elevated concentration of alveolar macrophages and, to a lesser extent, lymphocytes, reflecting the development of the chronic phase of inflammation. The concentration of acute-phase cells (neutrophils, eosinophils, and monocytes) returned to baseline. On both the 8th and 22nd days following necropsy, the comparison drugs dexamethasone and pirfenidone did not alter the cellular composition compared to untreated animals (Table 9).

**Table 9:** BALF cytology.

	Group 1	Group 2	Group 3	Group 4
	Saline + Tween 80	BLM + Tween 80	BLM + dexamethasone	BLM + pirfenidone
	Mean ± SD (*N* = 6)	Mean ± SD (*N* = 6)	Mean ± SD (*N* = 6)	Mean ± SD (*N* = 6)
Day 8				
Total cell concentration ×10^5/mL	1.9 ± 0.7	4.4 ± 2.0*	4.5 ± 1.3**	4.4 ± 2.1**
Alveolar macrophages, %	98 ± 2	46 ± 22**	48 ± 10**	47 ± 23**
Alveolar macrophages, ×10^5/mL	1.8 ± 0.7	1.8 ± 0.7	2.1 ± 0.6	2.2 ± 1.9
Band neutrophils,%	0.1 ± 0.1	22 ± 10**	30 ± 8**	26 ± 13**
Band neutrophils, ×10^5/mL	0.0 ± 0.0	1.1 ± 0.7**	1.3 ± 0.5**	1.0 ± 0.7**
Segmented neutrophils, %	0.0 ± 0.0	0.1 ± 0.1	0.1 ± 0.2	0.0 ± 0.0
Segmented neutrophils, ×10^5/mL	0.0 ± 0.0	0.0 ± 0.0	0.0 ± 0.0	0.0 ± 0.0
Eosinophils, %	0.1 ± 0.2	15 ± 10**	9 ± 5**	12 ± 8**
Eosinophils, ×10^5/mL	0.0 ± 0.0	0.7 ± 0.5**	0.4 ± 0.3**	0.5 ± 0.3**
Lymphocytes,%	2.2 ± 1.3	13 ± 3**	12 ± 3**	13 ± 6**
Lymphocytes, ×10^5/mL	0.0 ± 0.0	0.6 ± 0.3**	0.6 ± 0.3**	0.6 ± 0.6**
Monocytes,%	0.0 ± 0.0	3.7 ± 2.3**	1.5 ± 0.9**	2.2 ± 1.4**
Monocytes, ×10^5/mL	0.0 ± 0.0	0.2 ± 0.2**	0.1 ± 0.1**	0.1 ± 0.1**
Day 22				
Total cell concentration ×10^5/mL	1.5 ± 0.3	6.7 ± 1.5**	6.1 ± 1.8**	5.4 ± 1.4**
Alveolar macrophages, %	98.0 ± 0.9	96.7 ± 1.5	90 ± 15**	93 ± 2**
Alveolar macrophages, ×10^5/mL	1.4 ± 0.2	6.5 ± 1.4**	5. 7 ± 2.1**	5.0 ± 1.4**
Band neutrophils,%	0.1 ± 0.2	0.7 ± 0.6	4.0 ± 7.2**	2.3 ± 1.2**
Band neutrophils, ×10^5/mL	0.0 ± 0.0	0.1 ± 0.0	0.2 ± 0.2**	0.1 ± 0.1**
Segmented neutrophils, %	0.0 ± 0.0	0.0 ± 0.0	0.1 ± 0.1	0.0 ± 0.0
Segmented neutrophils, ×10^5/mL	0.0 ± 0.0	0.0 ± 0.0	0.0 ± 0.0	0.0 ± 0.0
Eosinophils, %	0.0 ± 0.0	0.1 ± 0.1	1.5 ± 3.2	0.4 ± 0.3
Eosinophils, ×10^5/mL	0.0 ± 0.0	0.0 ± 0.0	0.1 ± 0.1	0.0 ± 0.0
Lymphocytes, %	1.8 ± 0.9	2.5 ± 1.1	4.5 ± 4.5	3.9 ± 0.9**
Lymphocytes, ×10^5/mL	0.0 ± 0.0	0.2 ± 0.1**	0.2 ± 0.1**	0.2 ± 0.0**
Monocytes,%	0.0 ± 0.0	0.0 ± 0.0	0.1 ± 0.3	0.3 ± 0.4
Monocytes, ×10^5/mL	0.0 ± 0.0	0.0 ± 0.0	0.0 ± 0.0	0.0 ± 0.0

* ≤ 0.05, ** ≤ 0.01 relative to group 1, which received Saline + Tween 80, in paired comparison using the Mann–Whitney U test.

### 3.8 Hydroxyproline ELISA

**Figure 6:**
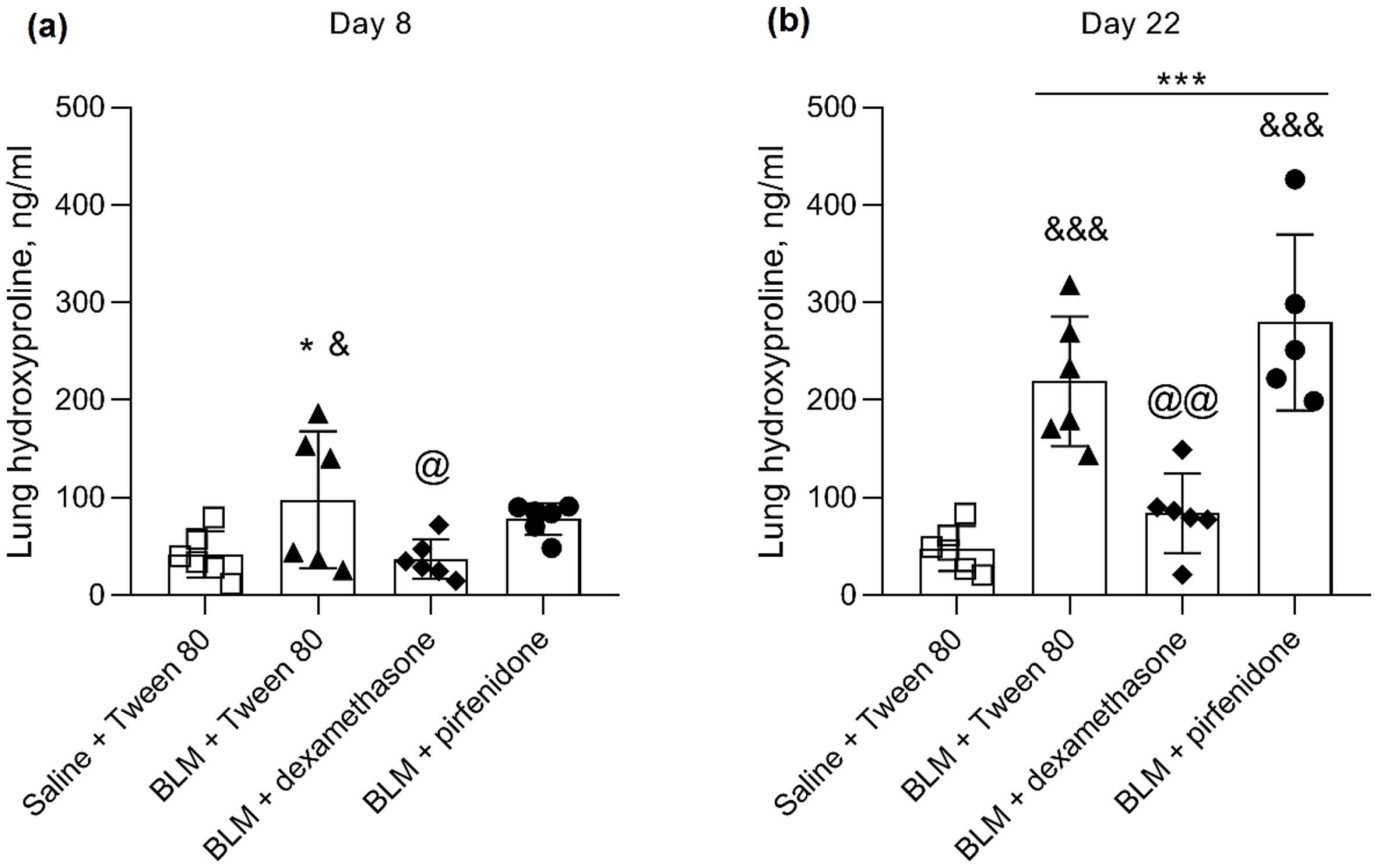
Hydroxyproline concentration in lung sample homogenates at necropsy on day 8 **(a)** and day 22 **(b)**. *p ≤ 0.05, ***p ≤ 0.001 relative to group 1 Saline + Tween 80, @ *p* ≤ 0.05, @@ *p* ≤ 0.01 relative to group 2 BLM + Tween 80, & *p* ≤ 0.05, &&& *p* ≤ 0.001 relative to group 3 BLM + dexamethasone according to Mann–Whitney U-test. In all groups for day 8 *n* = 12, for day 22 *n* = 6, except for group 4 (BLM + pirfenidone), where one animal died on day 9 of the study, and *n* = 5.

On the 8th day, the concentration of hydroxyproline in all groups receiving BLM intratracheally increased relatively to group 1, which received saline intratracheally. The exception were animals from group 3, which received BLM + dexamethasone, in which the hydroxyproline level was not only increased compared to animals from the control group1, but was also significantly lower in comparison with animals from group 2, which received BLM + Tween 80. On the 22nd day, the concentration of hydroxyproline was significantly higher in all groups that received intratracheal BLM compared to animals from group 1, which received intratracheal saline. In group 3, which received BLM + dexamethasone, the concentration of hydroxyproline was significantly lower compared to groups 2 and 4, which received BLM + Tween 80 and BLM + pirfenidone, respectively (Figure 6). Thus, pirfenidone had no effect on lung hydroxyproline levels, while dexamethasone significantly lowered them compared to both untreated and pirfenidone groups.

### 3.9 Blood biochemistry

The biochemical analysis data are presented in Table 10. On the 8th day post the PF induction, a decrease in ALP, albumin, calcium, inorganic phosphates and total protein was observed in animals without treatment. The same changes were also observed in the group of animals receiving pirfenidone. In animals receiving dexamethasone, the concentration of triglycerides increased 2.5 times compared to the control, and the concentration of total bilirubin increased. Biochemical analysis performed on the 8th day after the PF modeling showed that dexamethasone counteracted the decrease in albumin, calcium, inorganic phosphates, and total protein, but not alkaline phosphatase, which, as in other animals with PF (untreated and treated with pirfenidone), decreased by 1.5 times compared to the control. At necropsy on the 22nd day after the PF modeling, all animals with PF showed decreased concentrations of urea, AST, and creatinine. Level of alkaline phosphatase, calcium, inorganic phosphates, albumin, and total protein returned to baseline by the 22nd day. However, in animals treated with pirfenidone, albumin and total protein concentrations did not normalize, remaining lower even compared to untreated animals.

**Table 10:** Blood biochemistry.

	Group 1	Group 2	Group 3	Group 4
	Saline + Tween 80	BLM + Tween 80	BLM + dexamethasone	BLM + pirfenidone
	Mean ± SD (*N* = 6)	Mean ± SD (*N* = 6)	Mean ± SD (*N* = 6)	Mean ± SD (*N* = 6)
Day 8				
Urea, mmol/L	7.8 ± 0.9	7.1 ± 1.0	7.6 ± 0.9	7.5 ± 2.6
Cholesterol, mmol/L	2.7 ± 0.4	3.1 ± 0.8	2.6 ± 0.3	2.9 ± 0.8
Triglycerides, mmol/L	0.7 ± 0.2	0.6 ± 0.3	1.8 ± 1.0*	0.6 ± 0.1
ALT, U/L	64 ± 11	49 ± 13*	61 ± 7	59 ± 12
AST, U/L	89 ± 6	88 ± 14	99 ± 10	93 ± 29
Total bilirubin, μmol/L	3.4 ± 0.5	4.0 ± 1.3	5.6 ± 1.1**	4.1 ± 1.1
Creatinine, μmol/L	62 ± 5	59 ± 1	70 ± 10	57 ± 3
ALP, U/L	760 ± 157	438 ± 114*	488 ± 118*	372 ± 87**
Albumin, g/L	37.1 ± 1.5	34.2 ± 0.9*	39.9 ± 2.8	33.9 ± 1.0**
Calcium, mmol/L	2.8 ± 0.2	2.5 ± 0.1*	2.7 ± 0.2	2.5 ± 0.1*
Inorganic phosphates, mmol/L	2.8 ± 0.2	2.4 ± 0.2*	2.7 ± 0.2	2.4 ± 0.2*
Total protein, g/L	64 ± 4	58 ± 2*	66 ± 5	58 ± 2*
Day 22				
Urea, mmol/L	8.6 ± 0.5	6.8 ± 0.8*	6.7 ± 0.4**	7.1 ± 1.2*
Cholesterol, mmol/L	2.5 ± 0.5	2.8 ± 0.3	3.1 ± 0.2	2.7 ± 0.4
Triglycerides, mmol/L	0.8 ± 0.2	0.8 ± 0.1*	0.9 ± 0.2	0.9 ± 0.1
ALT, U/L	64 ± 12	59 ± 9	59 ± 12	70 ± 13
AST, U/L	111 ± 17	91 ± 8*	90 ± 9*	88 ± 9*
Total bilirubin, μmol/L	3.8 ± 1.0	3.6 ± 0.5	3.2 ± 0.5	3.7 ± 0.5
Creatinine, μmol/L	70 ± 16	62 ± 7*	64 ± 6*	59 ± 3*
ALP, U/L	771 ± 176	774 ± 155	728 ± 156	731 ± 70
Albumin, g/L	42 ± 5	37 ± 3	37 ± 3	36 ± 2*
Calcium, mmol/L	2.9 ± 0.2	2.7 ± 0.3	2.9 ± 0.3	2.7 ± 0.1
Inorganic phosphates, mmol/L	2.7 ± 0.4	2.6 ± 0.3	2.8 ± 0.3	2.7 ± 0.1
Total protein, g/L	69 ± 6	65 ± 7	63 ± 5	60 ± 3*

* ≤ 0.05, ** ≤ 0.01 relative to group 1, which received Saline + Tween 80, in paired comparison using the Mann–Whitney U test.

### 3.10 Histology

**Figure 7:**
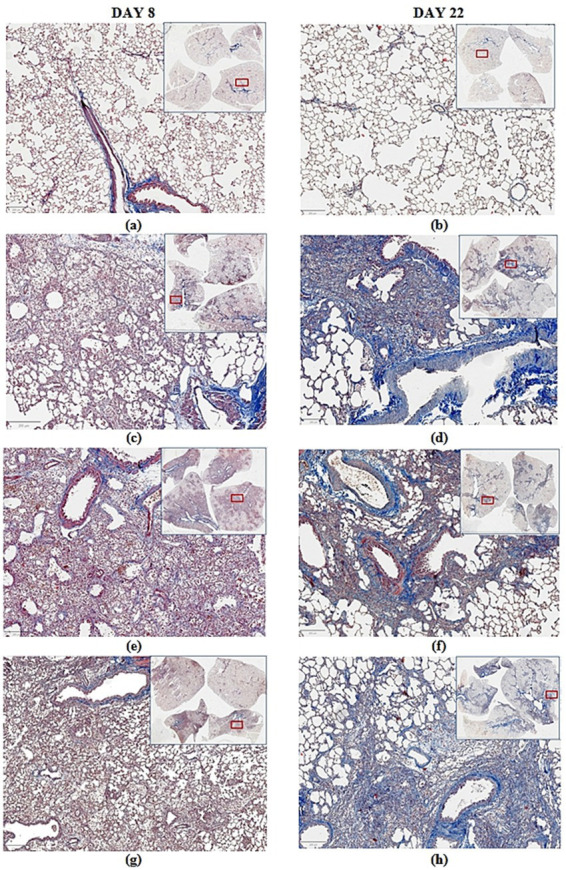
Photomicrographs of lung sections stained with Masson’s trichrome. The sections are presented as scanned images of all lobes of the right lung against the background of an enlarged fragment, the location of which is indicated by a red rectangle. Each row shows paired sections from each group of animals: on the left—sections from animals euthanized on day 8, on the right—sections from animals euthanized on day 22. **(a,b)** Paired sections from control animals treated with saline + Tween 80; **(c,d)** paired sections from animals treated with BLM + Tween 80; **(e,f)** paired sections from animals treated with BLM + dexamethasone; **(g,h)** paired sections from animals treated with BLM + pirfenidone. Digital images were obtained by scanning slides using a digital pathology slide scanner KF-PRO-005-HI (Ningbo Konfoong Bioinformation Tech Co., Ltd., China) at magnification ×40.

In the lungs of animals, euthanized on 8th day after PF modeling, inflammatory phenomena prevailed, which were characterized by large-focal hilar peribronchial location in all examined lobes of right lung with pronounced mononuclear infiltration of alveoli walls and lumen, areas of dystelectasis, transudate in lumen of acini. Fibrous changes at this observation period were insignificant, initial. The Ashcroft scale was not applicable at this time stage, and only a 5-point scale of pathomorphological deviations was used (Table 11). In all groups of animals received BLM intratracheally, signs of inflammation were observed in examined right lung, severity of which was minimal with dexamethasone administration—3.2 ± 0.4 points. A decrease of dystelectasis areas in this group indicates more active participation of lungs in systemic gas exchange. When assessing lung damage in animals euthanized on the 22nd day after PF induction, both a 5-point scale of pathomorphological changes (for preparations stained with hematoxylin and eosin) and a modified 8-point Ashcroft scale for assessing the severity of fibrosis (for preparations stained with Masson’s trichrome) were used (Table 12). Focal signs of PF were observed in the examined right lung of animals that received BLM intratracheally. Fibrous tissue foci were most frequently localized in peribronchial spaces of lung hilum, less frequently in the pulmonary acini without obvious connection with bronchi and, in isolated cases, subpleurally (Figure 7). The average fibrous tissue score according to the semi-quantitative 8-point modified Ashcroft scale in the group of animals receiving Tween 80 was 3.6 ± 0.8. Administration of dexamethasone did not produce an evident positive effect (3.4 ± 1.1 points), although the average fibrous tissue score in this group was minimal. In the group of animals receiving the second comparison drug, pirfenidone, the average fibrous tissue severity score was maximum—4.2 ± 0.1 points. Thus, administration of the comparator drug dexamethasone did not significantly affect the development of fibrotic fibrosis, although a minimal trend toward a reduction in the severity of fibrotic changes, as measured by the mean semiquantitative assessment score, was observed. The comparator drug pirfenidone demonstrated the worst effect among all study groups.

**Table 11:** Semi-quantitative histological assessment of the lungs on day 8.

Group number	Group name	Deviation assessment on a standard scale (0–5 points)	
		Mean ± SD	*N*
1	Saline + Tween 80	0.0 ± 0.0	6
2	BLM + Tween 80	3.7 ± 1.0*	6
3	BLM + dexamethasone	3.2 ± 0.4*	6
4	BLM + pirfenidone	4.1 ± 0.8*	6

*p ≤ 0.05 relative to group 1 according to Mann–Whitney U test for pairwise comparison.

**Table 12:** Semi-quantitative histological assessment of the lungs on day 22.

Group number	Group name	Deviation assessment on a standard scale (0–5 points)		Modified Ashcroft fibrosis score (0–8 points)	
		Mean ± SD	*N*	Mean ± SD	*N*
1	Saline + Tween 80	0.0 ± 0.0	6	0.1 ± 0.0	6
2	BLM + Tween 80	3.1 ± 1.0*	6	3.6 ± 0.8*	6
3	BLM + dexamethasone	3.0 ± 1.3*	6	3.4 ± 1.1*	6
4	BLM + pirfenidone	4.0 ± 0.0*	5	4.2 ± 0.1*	5

*p ≤ 0.05 relative to group 1 according to Mann–Whitney U test for pairwise comparison.

## 4 Discussion

At the early stage following lung injury, approximately 1 week post-injury, there is an increase in the number of proinflammatory M1 macrophages within the lung tissue. These cells secrete a variety of proinflammatory cytokines, including TNF, IL-1β, IL-6, and NO, as well as growth factors such as M-CSF and GM-CSF (48–51). Over time, these M1 macrophages are transformed into anti-inflammatory, profibrogenic M2 macrophages (52, 53), which then secrete growth factors including GF-β, fibroblast growth factor (FGF), platelet-derived growth factor-α (PDGFα), insulin-like growth factor 1 (IGF1), and vascular endothelial growth factor (VEGF) (54). Functionally, M2 macrophages are responsible for promoting tissue remodeling, inducing fibrosis, supporting angiogenesis and vasculogenesis, and even facilitating tumor progression (55–57). In rats, this initial inflammatory stage persists for about 7 days, after which the process of fibrosis formation begins, with fully developed fibrosis typically present by day 21. Accordingly, two necropsy periods were selected for this study: the 8th and 22nd days post-injury.

During the inflammatory phase, analysis of BALF revealed a dramatic increase in band neutrophils, with their relative number rising by 500 times on the 8th day after PF modeling compared to control animals. Proinflammatory chemokines produced by macrophages, which are the predominant cell type in BALF, stimulate increased neutrophil migration. By the 21st day, neutrophil infiltration had decreased by an order of magnitude, but the relative content of neutrophils in BALF still remained 50 times higher than control levels. Similar trends are observed in patients with IPF, where both neutrophil and lymphocyte levels in BALF are elevated (58–60). Our study also demonstrated an increase in BALF lymphocyte content: sixfold during the acute inflammatory phase and 1.5–2 times during the fibrosis formation phase. Elevated neutrophil levels are known to contribute to fibrotic changes in the lung through the action of neutrophil elastase (NE), which is increased in PF patients (61) and affects the extracellular matrix of the pulmonary endothelium, thereby promoting fibrosis (62).

Neither dexamethasone nor pirfenidone altered the cellular composition of BALF in animals with PF, whether at the inflammatory stage or during the established fibrosis phase. Pirfenidone is effective clinically in IPF by improving pulmonary function, specifically by slowing the decline in forced vital capacity (FVC) by approximately 50% (21, 63) and reducing disease exacerbations (64). Although some clinical studies have reported improved 12-month survival for IPF patients treated with pirfenidone (65–67), long-term survival benefits have not been confirmed (32). As PF progresses, patients’ general conditions deteriorate, often resulting in significant weight loss and pulmonary cachexia (68, 69). In our study, treatment with pirfenidone and dexamethasone not only failed to improve the condition of animals with developing PF but also exacerbated weight loss, which was more pronounced than in untreated PF animals. This suggests that these drugs were not only ineffective in treating BLM-induced PF in animals but may have had a detrimental effect on their overall health.

External respiratory function, a critical parameter in clinical studies of pulmonary disease (70) but less frequently assessed in rodent models (71, 72), was evaluated in this study. Following PF modeling, animals exhibited increased respiratory rates and significantly decreased tidal volumes, consistent with patterns observed in human pulmonary disease, including PF (73, 74). Importantly, neither pirfenidone nor dexamethasone improved external respiratory function in these animals, despite positive effects reported for pirfenidone in human studies (21). Lung weight and the lung weight index (ratio of lung weight to body weight) were also assessed. After PF modeling, both parameters increased significantly relative to controls. Previous studies have shown that pirfenidone can reduce lung weight in rats (15, 37, 75) and, in some reports, in mice (76, 77). However, in this study, both pirfenidone and dexamethasone failed to affect lung weight or weight index at either the inflammatory (day 8) or fibrotic stages (day 22).

Blood biochemical analysis on day 8 post-PF modeling revealed that all animals administered BLM experienced decreases in alkaline phosphatase, albumin, calcium, inorganic phosphates, and total protein—changes likely representing BLM side effects. Notably, dexamethasone-treated animals exhibited a significant increase in triglycerides and total bilirubin, potentially indicative of drug-related side effects. By day 22, all animals treated with intratracheal BLM showed decreased levels of urea, creatinine, and AST, while animals receiving pirfenidone also demonstrated further reductions in albumin and total protein. Biochemical analysis results indicate a general deterioration in the animals’ health due to pulmonary fibrosis, which dexamethasone did not improve and pirfenidone even worsened.

Clinical studies in COVID-19 patients have shown that early dexamethasone therapy (6 mg/day intravenously, 10 days) does not affect the incidence or severity of PF (93). Similarly, animal studies using other fibrosis models, such as silicon-induced PF in mice, have found no benefit with long-term dexamethasone treatment. There was no effect of the anti-inflammatory treatment on lung inflammatory parameters as well as cytokine production (IL-10, IL-4, IL-13 and IFN-γ) in mice. Although in rats in the same study, a decrease in collagen levels in the lungs was observed (78). In mice, dexamethasone administration for 14 days, starting on day 1 after PF induction by intratracheal administration of BLM, resulted in a decrease in collagen mRNA expression (39). In this study, dexamethasone significantly decreased hydroxyproline concentration, a marker for collagen and tissue fibrosis, but this did not translate into improvements in general health or lung histology. Other studies have likewise reported that, aside from reducing hydroxyproline, dexamethasone does not improve other PF indicators (40, 79). Thus, although the hydroxyproline content is an indicator that indirectly indicates the amount of collagen in the tissue, it cannot be considered a key parameter in isolation from other parameters such as a semi-quantitative assessment of specifically stained lung sections and the general condition of the animals.

Although numerous studies have shown that pirfenidone reduces collagen and hydroxyproline content in the lung and improves histological characteristics of pulmonary fibrosis when used with comparable or higher doses of bleomycin (35, 75, 76, 80, 81), these effects were not observed in our study.

Histological evaluation using Masson trichrome staining revealed pronounced PF in all BLM-treated animals, with the most severe lung damage in those receiving pirfenidone. Despite a relatively large pool of data indicating pirfenidone efficacy in PF (see Table 1), no beneficial therapeutic effects were demonstrated in our study. Many studies focus on specific molecular or cellular factors, such as gene expression or interleukin levels, without evaluating the animals’ general condition—the most critical factor for disease prognosis. In this study, a comprehensive evaluation, including respiratory function, blood biochemistry, BALF cellular composition, histology, and general clinical signs, demonstrated that standard dosing regimens of dexamethasone and pirfenidone were ineffective in the BLM-induced PF model in SD rats.

A number of studies have demonstrated a decrease in hydroxyproline levels (76, 77, 81), a decrease in proinflammatory cytokines (15, 34, 75, 77, 82), but most studies did not evaluate the general condition of the animals, which has great prognostic significance. Thus, in the study of Xie et al. (76), although pirfenidone improved histological indices, reduced the concentration of hydroxyproline in the lungs and reduced the content of THF-α, it did not improve body weight loss in animals, indicating the development of pulmonary cachexia and an unfavorable general prognosis (67, 68).

One of the factors that may be responsible for lack of efficacy of pirfenidone in our study may be dosing frequency of the drug, since dosing frequency for humans is three times a day. However, after analyzing the studies listed in Table 1 that used pirfenidone as a comparator, we found that out of the 15 studies reviewed, 8 studies (15, 35, 36, 76, 77, 81–83) used pirfenidone as a once-daily drug, 7 studies (34, 37, 75, 80, 84–86) did not report the dosing frequency, and only one study (33) omitted to report that pirfenidone was used twice a day. Moreover, these studies did not reveal any patterns in the efficacy of pirfenidone in the PF model. Thus, the factor of pirfenidone dosing frequency does not seem to be significant for its efficacy as a comparator in studies modeling PF.

Theoretically, the rodent species used in PF modeling studies may influence efficacy of comparator drug. Of the 36 PF modeling studies in rodents that we analyzed and listed in Table 1, 19 were performed in rats (35–37, 41, 42, 79, 82, 84, 87–93), 16 in mice (4, 17, 33, 38–40, 76, 77, 80, 85, 86), and one in rats and mice (78). No significant species-specific differences in the efficacy of the comparator drugs dexamethasone and pirfenidone were observed.

## 5 Conclusion

A 21-day oral administration of pirfenidone at a dose of 50 mg/kg and a 7-day intravenous administration of dexamethasone at a dose of 0.5 mg/kg to male SD rats with PF model did not improve the animals’ condition; in fact, these drugs worsened some parameters. Researchers working with PF models should consider these results when planning experiments.

## Data Availability

The raw data supporting the conclusions of this article will be made available by the authors, without undue reservation.

## Footnotes

1
https://www.ncbi.nlm.nih.gov/books/NBK482130/

## Glossary

IPF: Idiopathic pulmonary fibrosis
PF: Pulmonary fibrosis
BLM: Bleomycin
FDA: Food and Drug Administration
BAL: Broncho-alveolar lavage
BALF: Broncho-alveolar Lavage Fluid
SOD: Superoxide dismutase
TGF: Transforming growth factor
IL: Interleukin
TNF: Tumor necrosis factor
IACUC: Institutional Animal Care and Use Committee
ELISA: Enzyme-linked immunosorbent assay
SD rats: Sprague–Dawley rats
SPF: Specific pathogen free
VEGF: Vascular endothelial growth factor
FGF: Fibroblast growth factor
GF: Growth factor-
M-CSF: Macrophage colony-stimulating factor
GM-CSF: Granulocyte-macrophage colony-stimulating factor
PDGF: Platelet-derived growth factor
IGF: Insulin-like growth factor
ALP: Alkaline phosphatase
AST: Aspartate aminotransferase
ALT: Alanine aminotransferase
